# Interactions Between Genetic Variants and Environmental Factors Affect Risk of Esophageal Adenocarcinoma and Barrett’s Esophagus

**DOI:** 10.1016/j.cgh.2018.03.007

**Published:** 2018-10

**Authors:** Jing Dong, David M. Levine, Matthew F. Buas, Rui Zhang, Lynn Onstad, Rebecca C. Fitzgerald, Stomach and Oesophageal Cancer Study (SOCS) Consortium, Douglas A. Corley, Nicholas J. Shaheen, Jesper Lagergren, Laura J. Hardie, Brian J. Reid, Prasad G. Iyer, Harvey A. Risch, Carlos Caldas, Isabel Caldas, Paul D. Pharoah, Geoffrey Liu, Marilie D. Gammon, Wong-Ho Chow, Leslie Bernstein, Nigel C. Bird, Weimin Ye, Anna H. Wu, Lesley A. Anderson, Stuart MacGregor, David C. Whiteman, Thomas L. Vaughan, Aaron P. Thrift

**Affiliations:** ∗ Section of Epidemiology and Population Sciences, Department of Medicine, Baylor College of Medicine, Houston, Texas; ‡ Dan L. Duncan Comprehensive Cancer Center, Baylor College of Medicine, Houston, Texas; § Department of Biostatistics, School of Public Health, University of Washington, Seattle, Washington; ‖ Division of Public Health Sciences, Fred Hutchinson Cancer Research Center, Seattle, Washington; ¶ Department of Cancer Prevention and Control, Roswell Park Cancer Institute, Buffalo, New York; # Medical Research Council Cancer Unit, Hutchison-MRC Research Centre, University of Cambridge, Cambridge, United Kingdom; ∗∗ Division of Research, Kaiser Permanente Northern California, Oakland, California; ‡‡ San Francisco Medical Center, Kaiser Permanente Northern California, San Francisco, California; §§ Division of Gastroenterology and Hepatology, University of North Carolina School of Medicine, Chapel Hill, North Carolina; ‖‖ Department of Molecular Medicine and Surgery, Karolinska Institutet, Stockholm, Sweden; ¶¶ School of Cancer Sciences, King’s College London, London, United Kingdom; ## Division of Epidemiology, LICAMM, School of Medicine, University of Leeds, Leeds, United Kingdom; ∗∗∗ Division of Gastroenterology and Hepatology, Mayo Clinic, Rochester, Minnesota; ‡‡‡ Department of Chronic Disease Epidemiology, Yale School of Public Health, New Haven, Connecticut; §§§ Cancer Research UK, Cambridge Institute, Cambridge, United Kingdom; ‖‖‖ Department of Oncology, University of Cambridge, Cambridge, United Kingdom; ¶¶¶ Department of Public Health and Primary Care, University of Cambridge, Cambridge, United Kingdom; ### Pharmacogenomic Epidemiology, Ontario Cancer Institute, Toronto, Canada; ∗∗∗∗ Department of Epidemiology, University of North Carolina, Chapel Hill, North Carolina; ‡‡‡‡ Department of Epidemiology, MD Anderson Cancer Center, Houston, Texas; §§§§ Department of Population Sciences, Beckman Research Institute and City of Hope Comprehensive Cancer Center, Duarte, California; ‖‖‖‖ Department of Oncology, Medical School, University of Sheffield, Sheffield, United Kingdom; ¶¶¶¶ Department of Medical Epidemiology and Biostatistics, Karolinska Institutet, Stockholm, Sweden; #### Department of Preventive Medicine, University of Southern California/Norris Comprehensive Cancer Center, Los Angeles, California; ∗∗∗∗∗ Centre for Public Health, Queen’s University Belfast, Belfast, United Kingdom; ‡‡‡‡‡ Statistical Genetics, QIMR Berghofer Medical Research Institute, Brisbane, Australia; §§§§§ Cancer Control, QIMR Berghofer Medical Research Institute, Brisbane, Australia

**Keywords:** Esophageal Neoplasm, Genetic Variants, Risk Factors, Esophagus, BE, Barrett’s esophagus, BMI, body mass index, CI, confidence interval, EA, esophageal adenocarcinoma, EAF, effect allele frequency, GERD, gastroesophageal reflux disease, GWAS, genome-wide association study, MAF, minor allele frequency, OR, odds ratio, SNP, single nucleotide polymorphism

## Abstract

**Background & Aims:**

Genome-wide association studies (GWAS) have identified more than 20 susceptibility loci for esophageal adenocarcinoma (EA) and Barrett’s esophagus (BE). However, variants in these loci account for a small fraction of cases of EA and BE. Genetic factors might interact with environmental factors to affect risk of EA and BE. We aimed to identify single nucleotide polymorphisms (SNPs) that may modify the associations of body mass index (BMI), smoking, and gastroesophageal reflux disease (GERD), with risks of EA and BE.

**Methods:**

We collected data on single BMI measurements, smoking status, and symptoms of GERD from 2284 patients with EA, 3104 patients with BE, and 2182 healthy individuals (controls) participating in the Barrett’s and Esophageal Adenocarcinoma Consortium GWAS, the UK Barrett’s Esophagus Gene Study, and the UK Stomach and Oesophageal Cancer Study. We analyzed 993,501 SNPs in DNA samples of all study subjects. We used standard case–control logistic regression to test for gene-environment interactions.

**Results:**

For EA, rs13429103 at chromosome 2p25.1, near the *RNF144A-LOC339788* gene, showed a borderline significant interaction with smoking status (*P =* 2.18×10^-7^). Ever smoking was associated with an almost 12-fold increase in risk of EA among individuals with rs13429103-AA genotype (odds ratio=11.82; 95% CI, 4.03–34.67). Three SNPs (rs12465911, rs2341926, rs13396805) at chromosome 2q23.3, near the *RND3-RBM43* gene, interacted with GERD symptoms (*P* = 1.70×10^-7^, *P* = 1.83×10^-7^, and *P* = 3.58×10^-7^, respectively) to affect risk of EA. For BE, rs491603 at chromosome 1p34.3, near the *EIF2C3* gene, and rs11631094 at chromosome 15q14, at the *SLC12A6* gene, interacted with BMI (*P* = 4.44×10^-7^) and pack-years of smoking history (*P* = 2.82×10^-7^), respectively.

**Conclusion:**

The associations of BMI, smoking, and GERD symptoms with risks of EA and BE appear to vary with SNPs at chromosomes 1, 2, and 15. Validation of these suggestive interactions is warranted.

Over the past 4 decades, the incidence of esophageal adenocarcinoma (EA) has increased markedly in many Western populations. Among white men in the United States the incidence has increased almost 10-fold,^1^ and rates continue to rise by 2% per year.^2^ EA is a highly fatal cancer with a median overall survival of <1 year following diagnosis.^3^ EAs typically arise on a background of a premalignant change in the lining of the esophagus known as Barrett’s esophagus (BE). Thus, proposals to prevent EA-associated morbidity and mortality have suggested focusing on identifying patients with BE and enrolling them in endoscopic surveillance programs, or on identifying and modifying risk factors for neoplastic progression.^4^, ^5^, ^6^

Epidemiologic studies have identified frequent or persistent symptoms of gastroesophageal reflux disease (GERD),^7^, ^8^ obesity,^9^ and smoking^10^, ^11^ as the principal factors associated with increased risks of EA and BE. These 3 factors together comprise almost 80% of the attributable burden of EA.^12^, ^13^ Genetic factors also influence risk of EA and BE. Recent genome-wide association studies (GWAS) and post-GWAS studies have identified more than 20 loci significantly associated with risks of EA and BE^14^; however, these variants seem to explain only a limited proportion of the heritability of these diseases (estimated to be 25% for EA and 35% for BE).^15^ It is possible that environmental risk factors for EA and BE may interact with multiple genes through various biological pathways to contribute to disease susceptibility. Given the strength of associations with known risk factors for EA and BE (especially when compared with most other cancers), and potentially shared biological pathways (eg, inflammation) underlying these risk factors,^16^ identifying gene-environment interactions may be more plausible in the setting of EA and BE. These gene-environment interactions may account for some of the missing heritability of EA and BE.^15^ However, previous efforts to identify gene-environment interactions for EA and BE have predominantly been candidate based and have involved only small numbers of single nucleotide polymorphisms (SNPs).^17^, ^18^, ^19^

With the aim of identifying SNPs that may modify the associations of body mass index (BMI), smoking, and GERD symptoms with risks of EA and BE, we used pooled questionnaire and genetic data from several studies to conduct a large scale genome-wide gene-environment interaction study of EA and BE.

## Methods

### Study Population

We obtained data from 1512 EA patients, 2413 BE patients, and 2185 control subjects of European ancestry from 14 epidemiologic studies conducted in Western Europe, Australia, and North America participating in the International Barrett’s and Esophageal Adenocarcinoma Consortium (http://beacon.tlvnet.net/) GWAS. The design of the Barrett’s and Esophageal Adenocarcinoma Consortium GWAS has been described in detail previously.^20^ Histological confirmation of EA and BE was carried out for all the participating studies. The pooled dataset also included an additional 1,003 EA patients and 882 BE patients from the United Kingdom Stomach and Oesophageal Cancer Study and the UK Barrett’s Esophagus Gene Study, respectively.^20^ The EA patients in the UK Stomach and Oesophageal Cancer Study had International Classification of Diseases coding of malignant neoplasm of the esophagus (C15) and pathological diagnosis of adenocarcinoma (M8140-8575). The BE patients were identified at endoscopy with confirmed histopathological diagnosis of intestinal metaplasia in the UK Barrett's Esophagus Gene Study. Each contributing study was performed under institutional review board approval and all participants gave informed consent.

### SNP Genotyping

Genotyping of buffy coat or whole blood DNA from all participants was conducted using the Illumina Omni1M Quad platform (San Diego, CA), in accordance with standard quality-control procedures.^21^ For quality control, genotyped SNPs were excluded based on call rate <95%, Hardy-Weinberg Equilibrium *P* value over controls of <10^–4^, or minor allele frequency (MAF) ≤2%. After quality assurance and quality control, 993,501 SNPs were used for the current analysis. The analysis was restricted to the subset of ethnically homogenous individuals of European ancestry (confirmed in GWAS samples using principal components analysis).^20^

### Environmental (“Exposure”) Variables

Individual-level exposure data for each study participant were harmonized and merged into a single deidentified dataset. The data were checked for consistency and completeness and any apparent inconsistencies were followed up with individual study investigators. Depending on the study, data from self-reported written questionnaires or in-person interviews were obtained at or near the time of cancer diagnosis for EA patients, at or near the time of BE diagnosis for BE patients, and at the time of recruitment for control subjects. BMI was calculated as weight divided by square of height (kg/m^2^). For the analysis we selected the weight from each participant that likely reflected usual adult weight (before, for example, any disease-related weight loss). For tobacco smoking, the exposure variables were smoking status (ever vs never) and total cigarette smoking exposure among ever smokers (pack-years of smoking exposure). Ever cigarette smoking was defined as either low threshold exposure (≥100 cigarettes over their whole life) or by asking whether they had ever smoked regularly. Pack-years of smoking exposure was derived by dividing the average number of cigarettes smoked daily by 20 and multiplying by the total number of years smoked. GERD symptoms were defined as the presence of heartburn (ie, a burning or aching pain behind the sternum) or acid reflux (ie, a sour taste from acid, bile, or other stomach contents rising up into the mouth). For analysis, we used the highest reported frequency for either GERD symptom. Participants were then categorized as recurrent vs not recurrent based on a frequency of weekly or greater GERD symptoms for “recurrent.”^7^ A total of 425 participants with missing values for all 3 covariates (BMI, smoking history, and history of GERD symptoms) were excluded from the analysis.

### Statistical Analysis

We used standard case-control logistic regression to test for gene-environment interactions. SNP genotypes were treated as continuous variables and coded as 0, 1, or 2 copies of the minor allele. Exposure variables were either continuous (BMI and pack-years of smoking exposure) or dichotomous (smoking status and GERD symptoms). We modeled the gene-environment interaction by the product of the SNP genotype and the exposure variable, adjusting for age, sex, the first 4 principal components to control for possible population stratification, and the main terms of the SNP and the exposure variable. We used model-robust standard errors as suggested in Voorman et al^22^ to avoid inflated test statistics that can arise due to underestimation of variability in gene-environment GWAS. For SNPs from each of the top gene-environment interaction hits (ie, main text, *P* value for interaction <5.0 × 10^–7^) (Supplemental Material, *P* value for interaction <1.0 × 10^–6^) we also performed stratified analyses by genotype to examine the modified association of the known risk factor for EA or BE within the specific genotypes. Analyses were conducted using R software version 3.4.3. (R Foundation for Statistical Computing, Vienna, Austria), the GWASTools package,^23^ and Stata 13.0 (StataCorp LP, College Station, TX).

## Results

The final study sample included 2284 EA patients, 3104 BE patients, and 2182 control subjects. Characteristics of the study sample are shown in Table 1. On average, BMI was higher among EA (mean, 28.4 kg/m^2^) and BE (mean, 28.7 kg/m^2^) patients than among control subjects (mean, 27.0 kg/m^2^). Similarly, EA and BE patients were more likely than control subjects to be ever smokers (74.8%, 64.8%, and 59.1%, respectively) and to report history of recurrent GERD symptoms (46.9%, 52.9%, and 19.4%, respectively).

**Table 1:** Characteristics of the Study Population

Characteristic	Control Subjects n = 2182	EA n = 2284	Control Subjects vs EA *P* value^a^	BE n = 3104	Control Subjects vs BE *P* Value^a^
Age, y	61.7 ± 11.1	65.1 ± 10.3	<.001	62.9 ± 12.1	<.001
Sex			<.001		.008
Male	1715 (78.6)	1990 (87.1)		2343 (75.5)	
Female	467 (21.4)	294 (12.9)		761 (24.5)	
Body mass index, kg/m^2^			<.001		<.001
Mean	27.0 ± 4.7	28.4 ± 5.2		28.7 ± 5.1	
<25	786 (36.3)	245 (24.6)		608 (20.7)	
25–29.99	944 (43.5)	455 (45.8)		1191 (42.8)	
≥30	436 (20.2)	296 (29.6)		935 (36.5)	
Missing	16	1288		370	
Smoking status			<.001		<.001
Never	888 (40.9)	568 (25.2)		1081 (35.2)	
Ever	1282 (59.1)	1686 (74.8)		1994 (64.8)	
Missing	12	30		29	
Cumulative smoking history, pack-years^b^			.43		.001
Mean	32.8 ± 27.9	33.6 ± 26.4		29.4 ± 24.8	
Recurrent GERD symptoms			<.001		<.001
No	1446 (80.6)	965 (53.1)		1058 (47.1)	
Yes	348 (19.4)	854 (46.9)		1186 (52.9)	
Missing	388	465		860	

*NOTE.* Values are mean ± SD or n (%).

BE, Barrett’s esophagus; EA, esophageal adenocarcinoma; GERD, gastroesophageal reflux disease.

a *P* value from chi-square tests for categorical variables and Student’s *t* test for continuous variables. Missing categories were excluded from comparison tests.

b Among ever smokers.

### Gene-Environment Interactions for EA

**Figure 1:**
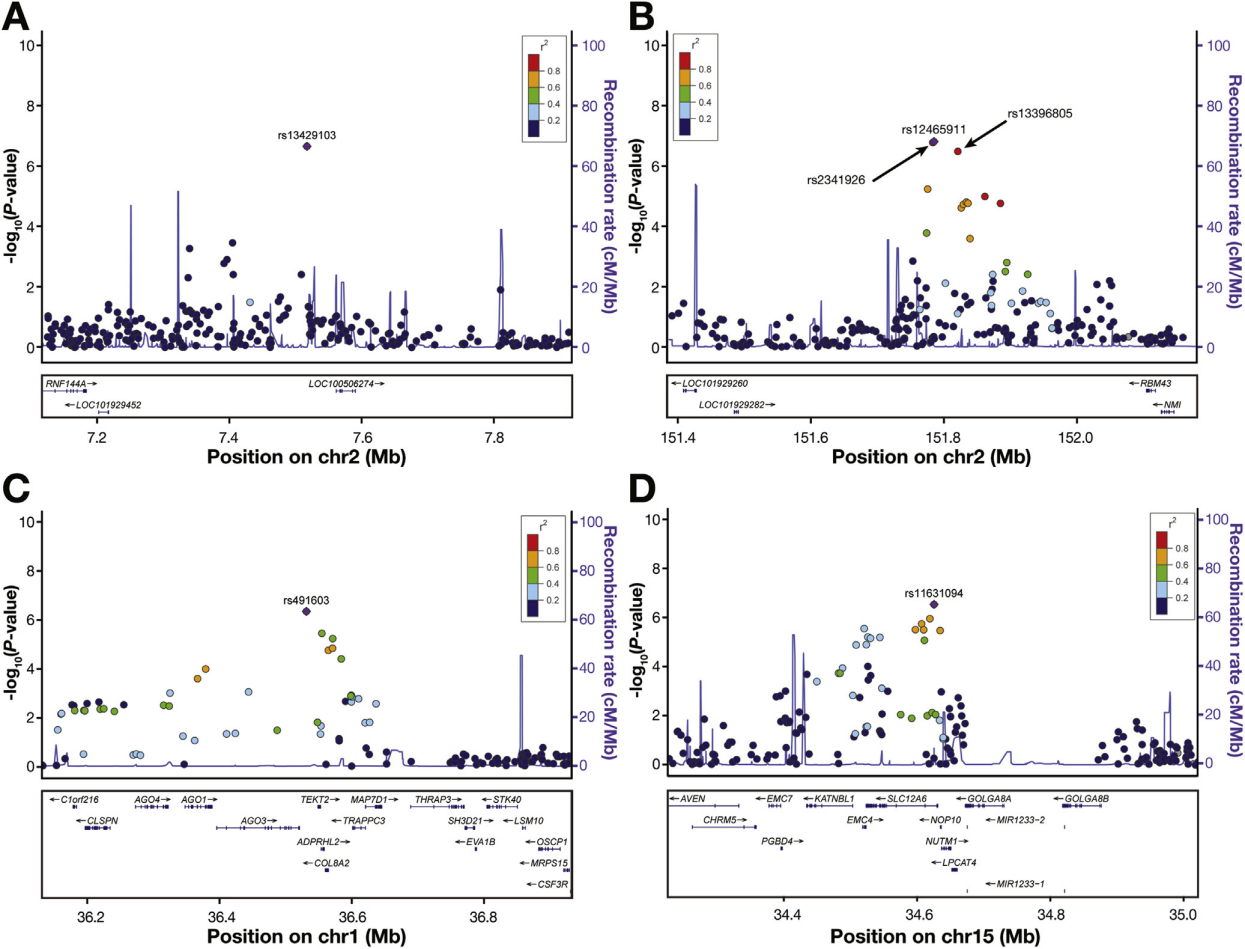
Regional association plots for genotyped single nucleotide polymorphisms (SNPs) showing *P* values for interaction for (*A*) smoking status and (*B*) recurrent gastroesophageal reflux disease symptoms in esophageal adenocarcinoma and (*C*) body mass index and (*D*) pack-years of smoking exposure in Barrett’s esophagus. The SNPs in Table 2 are shown as a *solid purple diamond*, except in panel *B* where rs2341926 and rs13396805 are shown as *circles* near rs12465911. The color scheme indicates linkage disequilibrium between the SNP shown with a *solid purple diamond* and other SNPs in the region using the *r*^2^ value calculated from the 1000 Genomes Project. The y axis is the −log10 interaction *P* value computed from 5388 cases (3104 Barrett’s esophagus, 2284 esophageal adenocarcinoma) and 2182 control subjects. The recombination rate from CEU HapMap data (right-side y axis) is shown in *light blue*. (*A*) Chromosome 2p25.1; (*B*) chromosome 2q23.3 region; (*C*) chromosome 1p34.3 region; (*D*) chromosome 15q14 region.

For EA, at borderline genome-wide significance, 1 SNP interacted with smoking status and 3 interacted with recurrent GERD symptoms (*P* for interactions ranging from 3.58 × 10^–7^ to 1.70 × 10^–7^) (Table 2, Figure 1*A* and *B*). At chromosome 2p25.1, rs13429103 (effect allele frequency [EAF] = 15.0%) showed interaction with smoking status (*RNF144A-LOC339788*, *P* = 2.18 × 10^–7^ for interaction). We also observed borderline statistically significant interactions between recurrent GERD symptoms and rs12465911 (*P* = 1.70 × 10^–7^ for interaction), rs2341926 (*P* = 1.83 × 10^–7^ for interaction), and rs13396805 (*P* = 3.58 × 10^–7^ for interaction) at chromosome 2q23.3 (*RND3-RBM43*). These 3 SNPs are in high linkage disequilibrium (all *r*^2^ > 0.9) as indicated in Figure 1*B*. Additional suggestive gene-environment interactions for EA (where *P <* 1.0 × 10^–6^ for interaction) are shown in Supplemental Table 1.

**Table 2:** Gene-Environment Interactions With EA or BE With a *P* Value for Interaction <5.0 × 10^–7^

Outcome	Exposure	SNP	Chr	Position	Gene	Effect/ Other	EAF	OR	*P*
EA									
	Smoking status	rs13429103	2p25.1	7517231	*RNF144A-LOC339788*	A/G	0.15	2.04	2.18 × 10^–7^
	Recurrent GERD symptoms	rs12465911	2q23.3	151785742	*RND3-RBM43*	A/G	0.26	2.03	1.70 × 10^–7^
	Recurrent GERD symptoms	rs2341926	2q23.3	151783928	*RND3-RBM43*	C/T	0.26	2.02	1.83 × 10^–7^
	Recurrent GERD symptoms	rs13396805	2q23.3	151821512	*RND3-RBM43*	A/G	0.26	1.99	3.58 × 10^–7^
BE									
	BMI (continuous)	rs491603	1p34.3	36532316	*EIF2C3-LOC100128093*	A/G	0.16	1.08	4.44 × 10^–7^
	Pack-years of smoking	rs11631094	15q14	34624438	*SLC12A6*	A/C	0.29	0.99	2.82 × 10^–7^

BE, Barrett’s esophagus; BMI, body mass index; EA, esophageal adenocarcinoma; EAF, effect allele frequency; GERD, gastroesophageal reflux disease; OR, odds ratio; SNP, single nucleotide polymorphism.

In analyses stratified by genotype (Table 3), compared with never smoking, ever smoking was associated with nearly a 12-fold higher risk of EA among individuals with rs13429103-AA genotype (odds ratio [OR], 11.82; 95% confidence interval [CI], 4.03–34.67). In contrast, among individuals with rs13429103-GG genotype, ever smoking conferred only 1.6-fold higher risk of EA (OR, 1.59; 95% CI, 1.36–1.85). Similarly, the risk for EA associated with recurrent GERD symptoms was higher in individuals with rs12465911-AA genotype (OR, 13.12; 95% CI, 6.21–27.73) than among individuals with rs12465911-GG genotype (OR, 2.80; 95% CI, 2.29–3.41). Additional stratified analyses for risk of EA are shown in Table 3 and Supplemental Table 2.

**Table 3:** Risk of EA and BE in Association With Obesity, Smoking History and Recurrent GERD Symptoms, Stratified by Genotype for SNPs in Table 2

Outcome	Environmental Exposure	SNP	Genotype	Cases/Control Subjects	OR	95% CI	*P* Value^a^
EA							
	Ever smoker vs never smoker (ref)	rs13429103	GG	1617/1572	1.59	1.36–1.85	<.001
			GA	589/554	2.91	2.23–3.81	<.001
			AA	48/44	11.82	4.03–34.67	<.001
	Recurrent GERD symptoms vs nonrecurrent GERD symptoms (ref)	rs12465911	GG	1206/1196	2.80	2.29–3.41	<.001
			GA	885/823	5.32	4.10–6.90	<.001
			AA	163/151	13.12	6.21–27.73	<.001
	Recurrent GERD symptoms vs nonrecurrent GERD symptoms (ref)	rs2341926	TT	975/985	2.80	2.30–3.42	<.001
			TC	724/681	5.30	4.08–6.88	<.001
			CC	120/128	13.12	6.21–27.73	<.001
	Recurrent GERD symptoms vs nonrecurrent GERD symptoms (ref)	rs13396805	GG	998/1005	2.85	2.34–3.48	<.001
			GA	701/662	5.23	4.02–6.81	<.001
			AA	120/127	12.73	6.12–26.49	<.001
BE							
	BMI ≥30 kg/m^2^ vs BMI <25 kg/m^2^ (ref)	rs491603	GG	1306/1137	1.52	1.38–1.67	<.001
			GA	438/518	2.11	1.80–2.47	<.001
			AA	42/64	3.30	1.90–5.73	<.001
	≥15 pack-years vs <15 pack-years (ref)	rs11631094	CC	729/618	1.02	0.81–1.30	.846
			CA	555/540	0.65	0.50–0.84	.001
			AA	115/106	0.52	0.28–0.95	.033

BE, Barrett’s esophagus; BMI, body mass index; CI, confidence interval; EA, esophageal adenocarcinoma; GERD, gastroesophageal reflux disease; OR, odds ratio; SNP, single nucleotide polymorphism.

a *P* values from logistic regression analysis adjusted for age and sex.

### Gene-Environment Interactions for BE

For BE, at chromosome 1p34.3, we observed an interaction between rs491603 (EAF = 16.5%) and BMI (*EIF2C3-LOC100128093*, *P* = 4.44 × 10^–7^ for interaction) (Table 2, Figure 1*C*). At chromosome 15p14, rs11631094 (EAF = 28.7%) showed interaction with pack-years of smoking exposure (*SLC12A6*, *P* = 2.82 × 10^–7^ for interaction) (Table 2, Figure 1*D*). Additional suggestive significant interactions (where *P <* 1.0 × 10^–6^ for interaction) for BE with pack-years of smoking exposure at chromosomes 12q23.1, 16p12.3, and 17q12 are presented in Supplemental Table 1.

Stratified analyses by genotype showed that the risk for BE associated with obesity (BMI ≥30 kg/m^2^) was elevated by over 200% among individuals with rs491603-AA genotype (vs BMI <25 kg/m^2^; OR, 3.30; 95% CI, 1.90–5.73) but only by approximately 50% among individuals with rs491603-GG genotype (vs BMI <25 kg/m^2^; OR, 1.52; 95% CI, 1.38–1.67). Additional stratified analyses of gene-environment interactions for BE are shown in Table 3 and Supplemental Table 2.

### Cross-Examination of Discovered Gene-Environment Interactions

For each SNP in Table 2 and Supplemental Table 1 that had a borderline significant genome-wide interaction in either EA or BE, we examined the equivalent gene-environment interaction in BE and EA, respectively (Supplemental Table 3). For all SNPs discovered in EA, we observed nominal levels of significance (*P* value for interaction <.05) and ORs in the same direction but somewhat attenuated in BE. For SNPs discovered in BE, only half had *P* value for interaction <.05 in EA, although all had similar ORs to those in BE. Although obesity and GERD are correlated, none of the SNPs with *P* value for interaction <1.0 × 10^–6^ with GERD had comparable ORs or *P* values when testing for interaction with obesity and similarly for the 1 obesity SNP when tested for GERD.

## Discussion

To our knowledge, this is the first genome-wide gene-environment interaction study of EA and its precursor, BE. Although no gene-environment interactions reached genome-wide significance (ie, *P <* 5.0 × 10^–8^ for interaction), several borderline significant interactions were indicated between SNPs and known risk factors for EA and BE – BMI, smoking, and GERD symptoms.

A number of studies have pursued candidate-based gene-environment analyses of EA, and reported interactions between BMI, smoking or GERD symptoms and selected SNPs in genes related to detoxification, angiogenesis, DNA repair, apoptosis, and extracellular matrix degradation.^24^, ^25^, ^26^, ^27^, ^28^, ^29^, ^30^, ^31^ This body of work helped to establish the notion that the level of disease risk associated with GERD symptoms, in particular, may vary according to inherited genetic variation. All of these studies, however, were conducted in small samples (<350 cases) and were not replicated in independent populations. While direct comparison of our own results and these past findings is complicated by less-than-complete overlap of genotyped SNPs between studies, we did not find evidence in support of interactions among BMI, smoking, or GERD symptoms and any assessed variants in previously-implicated genes: *GSTM1*, *GSTT1*, *VEGF*, *MGMT*, *EGF*, *IL1B*, *PERP*, *PIK3CA*, *TNFRSF1A*, *CASP7*, *TP53BP1*, *BCL2*, *HIF1AN*, *PDGRFA*, *VEGFR1*, or *MMP1* (Supplemental Table 4). It remains possible that nominal evidence for some of these associations may not have survived stringent correction for multiple comparisons, and larger samples are needed for true signals to reach significance. Alternatively, previously reported interactions may simply reflect chance findings in small samples because they did not validate in our large study population.

This study has several strengths. First, the pooled dataset including relatively large numbers of cases and control subjects provided us with a rare opportunity to perform, in parallel, genome-wide gene-environment interaction analyses for EA and its precursor lesion, BE. Past candidate-based gene-environment interaction studies of EA have focused on small numbers of genes selected according to biological plausibility, and collectively these reports sampled only a small fraction of the total SNPs presently analyzed (N = 993,501). Such preconceived “gene-centric” SNP selection methods fail to capture the large fraction of noncoding intergenic variations that have been linked to altered risk for these 2 conditions, and also artificially restricts the “genic” search space based on limited mechanistic knowledge, a limitation that is overcome by an unbiased comprehensive genome-wide gene-environment interaction assessment. Second, our study draws on genetic and epidemiologic data from a recent consortium-based GWAS of EA/BE,^20^ which is the largest of its kind. This sizable study sample afforded greater power to detect gene-environment interactions than in any previous study. Third, all genotyping from this GWAS was conducted on a single platform and in a single laboratory, and subjected to stringent quality-control procedures. Most GWAS analyses test only an additive model because an additive model has reasonable power to detect both additive and dominant effects and the 2 models yield similar results and many GWAS analyses, including ours, are underpowered to detect recessive effects. Nevertheless, for completeness we also tested a dominant model for the 16 SNPs with a *P* value for interaction <1.0 × 10^–6^ (Table 2 and Supplemental Table 1), and found slightly attenuated results of the ORs for some gene-environment interactions (data not shown).

Our study also has some limitations. First, our ability to detect true gene-environment interactions might have been limited by the manner in which the environmental (exposure) variables were measured and harmonized. For example, recall bias is a possibility during retrospective reporting of the exposures in the parent case-control studies. However, respondents were unaware of their genotype status at the time of the interviews, mitigating the impact of any possible recall bias in our interaction analyses. Similarly, while considerable care was taken during data harmonization, as described in a series of recent pooled analyses,^10^, ^11^ some potential for measurement error of the exposures examined is possible. However, given that case-control status was not considered during this process, any errors from harmonization would be nondifferential, resulting in attenuation of the resulting ORs. Second, central obesity (eg, waist-to-hip ratio) has been found to be more strongly associated with the risk of BE than BMI; however, as waist and hip measurements were not collected in the majority of the included studies, we were unable to examine for interactions with central obesity. Third, despite the comprehensive nature of the genome-wide analysis, we were nonetheless limited to examining common genetic variation (MAF >2%) represented on the Illumina Omni1M Quad GWAS platform employed. Further large-scale studies based on whole-exome or whole-genome sequencing would be required to identify additional gene-environment interactions with rare variants, and more precisely map the reported associations. Finally, our study results should be considered as discovery findings, worthy of independent replication. None of the interactions studied reached genome-wide significance (ie, *P <* 5.0 × 10^–8^ for interaction). This may be because there are truly no gene-environment interactions or it may be that power was still limited to detect modest or weak interactions despite our large sample size. In our analyses of 2284 EA patients, 3104 BE patients, and 2182 control subjects, we were adequately powered to detect interactions with an interaction OR in the range of 1.98–2.52 for MAF in the observed range (0.11–0.43), assuming a main effect of 1.08 for log-additive SNPs, a main effect of 1.90 for binary risk factors, and an α of 5.0 × 10^–8^. Given the large worldwide consortia sample of patients participating in this work, few additional studies of EA and BE patients are currently available and have data for replication; thus, such work may require additional time for study patients to accrue.

In conclusion, our report describes the first genome-wide gene-environment interaction analysis for EA and BE. These findings provide evidence that the magnitude of disease risk associated with BMI, smoking, and GERD symptoms may differ according to germline genetics, and suggest the potential utility of combing epidemiologic exposure data with selected genotyping for comprehensive risk assessment in patients susceptible to EA or BE. Pending validation of the observed interactions in independent study populations, further analyses will be required to investigate the biological basis for differential disease risk associated with the risk factors investigated in the presence of these variants.

## Figures and Tables

**Supplemental Table 1:** Gene-Environment Interactions With EA or BE With a *P* Value for Interaction <1.0 × 10^–6^

Outcome	Exposure	SNP	Chr	Position	Gene	Effect/ Other	EAF	OR	*P* Value
EA									
	Smoking status	rs2434584	5q11.2	57566073	*ACTBL2-PLK2*	C/T	0.08	2.52	7.44 × 10^–7^
	Smoking status	rs40210	5q11.2	57619964	*ACTBL2-PLK2*	A/G	0.08	2.46	8.82 × 10^–7^
	Pack-years of smoking	rs17002540	Xq27.1	139946061	*CDR1-SPANXB2*	T/C	0.19	0.99	5.92 × 10^–7^
	Recurrent GERD symptoms	rs2971030	7p21.3	10006341	*LOC340268*	G/A	0.42	1.77	6.02 × 10^–7^
	Recurrent GERD symptoms	rs7141987	14q32.31	101492224	*SNORD114-31-LOC100130814*	G/A	0.42	1.77	7.11 × 10^–7^
	Recurrent GERD symptoms	rs2971028	7p21.3	10007255	*LOC340268*	A/G	0.40	1.76	8.56 × 10^–7^
									
BE									
	Pack-years of smoking	rs9668109	12q23.1	99011272	*IKIP*	A/G	0.09	0.98	6.31 × 10^–7^
	Pack-years of smoking	rs1548445	16p12.3	19691583	*C16orf62*	G/A	0.06	1.02	8.21 × 10^–7^
	Pack-years of smoking	rs2671828	17q12	33731764	*SLFN11-LOC729839*	A/G	0.43	0.99	9.54 × 10^–7^
	Pack-years of smoking	rs10507102	12q23.1	98990871	*SLC25A3*	A/G	0.09	0.98	9.91 × 10^–7^

BE, Barrett’s esophagus; BMI, body mass index; EA, esophageal adenocarcinoma; EAF, effect allele frequency; GERD, gastroesophageal reflux disease; OR, odds ratio; SNP, single nucleotide polymorphism.

**Supplemental Table 2:** Risk of EA and BE in Association With Smoking History and GERD Symptoms, Stratified by Genotype for SNPs in Supplemental Table 1

Outcome	Environmental Exposure	SNP	Genotype	Cases/Control Subjects	OR	95% CI	*P* Value^a^
EA							
	Ever smoker vs never smoker (ref)	rs2434584	TT	1907/1826	1.67	1.45–1.93	<.001
			CT	342/328	4.33	3.00–6.24	<.001
			CC	5/15	NA	-	-
							
	Ever smoker vs never smoker (ref)	rs40210	GG	1903/1821	1.67	1.45–1.92	<.001
			GA	344/332	4.24	2.96–6.06	<.001
			AA	6/16	NA	-	-
							
	≥15 pack-years vs <15 pack-years (ref)	rs17002540	CC	1053/1003	1.36	1.12–1.66	.002
			CT	48/55	0.78	0.33–1.86	.579
			TT	218/206	0.63	0.39–1.00	.052
							
	Recurrent GERD symptoms vs nonrecurrent GERD symptoms (ref)	rs2971030	AA	599/603	2.68	2.08–3.44	<.001
			GA	908/895	3.81	3.06–4.75	<.001
			GG	309/293	9.44	6.17–14.45	<.001
							
	Recurrent GERD symptoms vs nonrecurrent GERD symptoms (ref)	rs7141987	AA	591/590	2.69	2.08–3.49	<.001
			GA	908/887	3.74	3.02–4.64	<.001
			GG	319/317	9.32	6.04–14.36	<.001
							
	Recurrent GERD symptoms vs nonrecurrent GERD symptoms (ref)	rs2971028	GG	625/635	2.70	2.11–3.45	<.001
			GA	900/890	3.87	3.10–4.82	<.001
			AA	294/268	9.58	6.17–14.88	<.001
							
							
BE							
	≥15 pack-years vs <15 pack-years (ref)	rs9668109	GG	1167/1058	0.92	0.77–1.11	.390
			GA	221/201	0.38	0.25–0.60	<.001
			AA	11/5	0.33	0.02–5.64	.443
							
	≥15 pack-years vs <15 pack-years (ref)	rs1548445	AA	1223/1097	0.76	0.63–0.91	.002
			GA	170/164	1.11	0.68–1.80	.675
			GG	6/3	NA	-	-
							
	≥15 pack-years vs <15 pack-years (ref)	rs2671828	GG	457/423	0.93	0.70–1.23	.595
			GA	688/588	0.84	0.66–1.07	.163
			AA	246/250	0.54	0.36–0.80	.002
							
	≥15 pack-years vs <15 pack-years (ref)	rs10507102	GG	1166/1058	0.93	0.77–1.11	.409
			GA	222/200	0.38	0.24–0.59	<.001
			AA	11/5	0.33	0.02–5.64	.443

BE, Barrett’s esophagus; BMI, body mass index; EA, esophageal adenocarcinoma; EAF, effect allele frequency; GERD, gastroesophageal reflux disease; SNP, single nucleotide polymorphism.

a *P* values from logistic regression analysis adjusted for age and sex.

**Supplemental Table 3:** Comparison of Gene-Environment Interactions in BE and EA for SNPs With *P* Value for Interaction <1.0 × 10^–6^ on the Outcomes

Exposure	SNP	Chr	Position	Gene	Effect/ Other	BE		EA	
						OR	*P*	OR	*P*
G × E hits for EA									
Smoking status	rs13429103	2p25.1	7517231	*RNF144A-LOC339788*	A/G	1.40	3.51 × 10^–3^	2.04	2.18 × 10^–7^
Smoking status	rs2434584	5q11.2	57566073	*ACTBL2-PLK2*	C/T	1.56	3.90 × 10^–3^	2.52	7.44 × 10^–7^
Smoking status	rs40210	5q11.2	57619964	*ACTBL2-PLK2*	A/G	1.54	4.94 × 10^–3^	2.46	8.82 × 10^–7^
Pack-years of smoking	rs17002540	Xq27.1	139946061	*CDR1-SPANXB2*	T/C	0.99	4.83 × 10^–3^	0.99	5.92 × 10^–7^
Recurrent GERD symptoms	rs12465911	2q23.3	151785742	*RND3-RBM43*	A/G	1.66	6.09 × 10^–5^	2.03	1.70 × 10^–7^
Recurrent GERD symptoms	rs2341926	2q23.3	151783928	*RND3-RBM43*	C/T	1.65	7.38 × 10^–5^	2.02	1.83 × 10^–7^
Recurrent GERD symptoms	rs13396805	2q23.3	151821512	*RND3-RBM43*	A/G	1.59	2.80 × 10^–4^	1.99	3.58 × 10^–7^
Recurrent GERD symptoms	rs2971030	7p21.3	10006341	*LOC340268*	G/A	1.36	5.03 × 10^–3^	1.77	6.02 × 10^–7^
Recurrent GERD symptoms	rs7141987	14q32.31	101492224	*SNORD114-31-LOC100130814*	G/A	1.29	1.40 × 10^–2^	1.77	7.11 × 10^–7^
Recurrent GERD symptoms	rs2971028	7p21.3	10007255	*LOC340268*	A/G	1.35	6.10 × 10^–3^	1.76	8.56 × 10^–7^
									
G × E hits for BE									
BMI (continuous)	rs491603	1p34.3	36532316	*EIF2C3-LOC100128093*	A/G	1.08	4.44 × 10^–7^	1.04	1.83 × 10^–2^
Pack-years of smoking	rs11631094	15q14	34624438	*SLC12A6*	A/C	0.99	2.82 × 10^–7^	1.00	0.125
Pack-years of smoking	rs9668109	12q23.1	99011272	*IKIP*	A/G	0.98	6.31 × 10^–7^	0.99	9.74 × 10^–3^
Pack-years of smoking	rs1548445	16p12.3	19691583	*C16orf62*	G/A	1.02	8.21 × 10^–7^	1.01	9.70 × 10^–2^
Pack-years of smoking	rs2671828	17q12	33731764	*SLFN11-LOC729839*	A/G	0.99	9.54 × 10^–7^	1.00	6.13 × 10^–2^
Pack-years of smoking	rs10507102	12q23.1	98990871	*SLC25A3*	A/G	0.98	9.91 × 10^–7^	0.99	6.93 × 10^–3^

BE, Barrett’s esophagus; BMI, body mass index; EA, esophageal adenocarcinoma; EAF, effect allele frequency; G × E, gene-environment; GERD, gastroesophageal reflux disease; OR, odds ratio; SNP, single nucleotide polymorphism.

**Supplemental Table 4:** Associations of Previously Reported Gene-Environment Interactions With Esophageal Adenocarcinoma in Our Study Population

Original Publication				Current Study	
Author	SNP	Exposure	*P* Value	Directly Genotyped or High-LD SNP	*P* Value
Casson et al, 2006^24^	NA	-	-	-	-
Zhai et al, 2008^25^	rs833061	Smoking	.03	rs833070	.068
Doecke et al, 2008^26^	rs12269324	GERD symptoms	-	Direct	.979
	rs12268840	GERD symptoms	-	Direct	.714
Cheung et al, 2009^27^	rs4444903	GERD symptoms	<.001^a^	Direct	.240
Zhai et al, 2012^28^	rs1143634	GERD symptoms	.008	Direct	.398
	rs1052486	BMI + Smoking	-	-	-
	rs1052486	BMI	-	Direct	.423
	rs1052486	Smoking	-	Direct	.532
Wu et al, 2011^29^	rs648802	GERD symptoms	.02	Direct	.838
	rs4855094	GERD symptoms	.04	Direct	.872
	rs7644468	GERD symptoms	.04	-	-
	rs4149579	GERD symptoms	.04	-	-
	rs560191	Smoking	.02	Direct	.331
	rs7907519	Smoking	.04	rs11196449	.868
	rs12454712	Smoking	.04	Direct	.435
Zhai et al, 2012^30^	rs2295778	GERD symptoms	.0005	rs12780796	.654
	rs13337626	GERD symptoms	.0067	rs34197769	.315
	rs2295778	Smoking	.004	-	-
	rs2296188	Smoking	.014	Direct	.905
	rs2114039	BMI	.0026	Direct	.228
	rs2296188	BMI	.0023	Direct	.452
	rs11941492	BMI	.013	Direct	NA^b^
	rs17708574	BMI	.013	Direct	.316
	rs7324547	BMI	.008	-	-
	rs17619601	BMI	.016	-	-
	rs17625898	BMI	.023	-	-
Cheung et al, 2012^31^	rs1799750	GERD symptoms	.002	-	-
	rs3025058	GERD symptoms	.04	-	-

BMI, body mass index; GERD, gastroesophageal reflux disease; LD, linkage disequilibrium; SNP, single nucleotide polymorphism.

a Two-way interaction.

b On array but quality control failure. We were unable to validate all SNPs as some were biallelic or we failed to identify a high LD SNP (*r*^2^ < 0.70).
